# 1T-ZrS_2_ Monolayer Decorated with Sc, Ti, and V Single Atoms: A Potential Gas Scavenger for NO_x_ and SO_2_

**DOI:** 10.3390/nano15211653

**Published:** 2025-10-29

**Authors:** Xiaoxuan Wang, Jiaqi Zhang, Jinjuan Zhang, Xiaoqing Liu, Yuanqi Lin, Fangfang Li, Guangwei Wang, Yan Xu, Peng Wang

**Affiliations:** 1 College of Electronic and Information Engineering, Shandong University of Science and Technology, Qingdao 266590, China; 202482130011@sdust.edu.cn (X.W.); lifangfang@sdust.edu.cn (F.L.);; 2 School of Mathematical Sciences, Shanghai Jiao Tong University, Shanghai 200240, China; liuxiaoq1994@gmail.com

**Keywords:** 1T-ZrS_2_ monolayer, single-atom decoration, gas scavenger, COHP

## Abstract

The intensification of industrialization and increasing energy consumption have led to elevated emissions of hazardous gases such as NO, NO_2_, and SO_2_, making their efficient capture and removal crucial for environmental remediation. In this work, first-principles calculations were employed to systematically investigate the adsorption behavior of these gases on single-atom-decorated (Sc, Ti, and V) 1T-ZrS_2_ monolayers. The results indicate that the transition metal atoms preferentially occupy the hexagonal hollow sites of ZrS_2_, forming an approximately octahedral coordination field and exhibiting characteristic *d*-orbital splitting. During gas adsorption, the decorated systems exhibit pronounced metal-to-adsorbate charge donation and strong *d*-*p* hybridization, indicative of strong chemisorption. Notably, Ti-ZrS_2_ exhibits the strongest adsorption toward NO_2_, inducing partial molecular dissociation and suggesting catalytic activity, whereas Sc- and V-decorated systems predominantly maintain molecular adsorption. Recovery time calculations indicate that the adsorption processes are comparatively stable, making these systems suitable for gas capture and pollution abatement. Overall, single-atom decoration provides an effective strategy to modulate the electronic structure and gas interactions of ZrS_2_, highlighting its potential as an efficient gas scavenger for NO, NO_2_, and SO_2_.

## 1. Introduction

With the intensification of industrialization and energy consumption, the emission of harmful gases such as nitrogen oxides NO_x_ (NO, NO_2_) and sulfur dioxide (SO_2_) into the atmosphere has continued to increase. These gaseous pollutants originate primarily from industrial combustion processes, vehicle exhaust emissions, and agricultural activities, which not only cause acid rain and photochemical smog but also pose serious threats to human health [1,2,3,4,5]. Therefore, developing efficient and stable gas sensing and capture materials is crucial for environmental remediation and the protection of human health [6,7,8,9].

Among various candidate materials, two-dimensional (2D) materials have attracted extensive attention owing to their high specific surface area, tunable electronic structures, and abundant active sites [10,11,12,13,14]. In particular, transition metal dichalcogenides (TMDCs), featuring layered crystal structures, adjustable band gaps, and excellent chemical stability, are considered ideal materials for the adsorption and immobilization of toxic gases [15,16,17,18,19,20,21,22]. In recent years, numerous studies have focused on gas capture applications based on monolayer TMDCs. For example, Cho et al. systematically investigated the adsorption behaviors of toxic gases such as O_3_, SO_2_, and SO_3_ on MoS_2_ monolayers through combined experimental and theoretical approaches, demonstrating the great potential of MoS_2_ for gas capture [23]. Similarly, Scardamaglia et al. experimentally synthesized WS_2_ monolayers and demonstrated their high-sensitivity detection capability toward NO_2_ molecules [24]. As a representative layered TMDC, ZrS_2_ has been successfully fabricated in various nanostructured forms, exhibiting good structural stability and semiconducting characteristics [25,26,27]. Benefiting from its excellent optical, thermal, and electronic properties, ZrS_2_ has been widely regarded as a promising candidate material for multifunctional applications [28,29,30]. For instance, Li et al. incorporated ZrS_2_ into erbium-doped fiber lasers, achieving high-performance dual-wavelength Q-switched pulse outputs [31].

Extensive first-principles studies have demonstrated that single-atom modification can significantly tune the surface reactivity and electronic properties of TMDCs [32,33,34,35,36,37,38]. For example, Zhang et al. systematically investigated Co-, Pd-, and Pt-decorated MoS_2_ monolayers and found that, compared with pristine MoS_2_, these systems exhibit remarkably enhanced adsorption capabilities toward small molecules such as NO_2_ and NH_3_ [39]. This enhancement mainly arises from the strong hybridization between the metal *d* orbitals and the molecular orbitals of the adsorbates. Likewise, Ni-decorated WSe_2_ monolayers exhibit significant modulation of electronic structures during CO and HCHO adsorption, indicating their potential as resistive-type gas sensing materials [40]. Pt- and Au-modified ZrSe_2_ systems have also shown improved chemical adsorption and gas-sensing performances [41]. Furthermore, several studies have extended the single-atom decoration strategy to ZrS_2_, revealing that transition metal decoration can significantly improve its gas adsorption and sensing behavior [42,43,44]. Notably, recent experimental advances have enabled the synthesis of several single-atom-modified TMDCs [45,46], which demonstrates the feasibility of preparing single-atom-decorated ZrS_2_.

Motivated by this, we employ first-principles calculations to systematically investigate the adsorption behavior of NO, NO_2_, and SO_2_ molecules on Sc-, Ti-, and V-decorated 1T-ZrS_2_ monolayers (1T denotes the trigonal phase of TMDCs with octahedral coordination). Sc, Ti, and V were selected as decorating atoms because they are located close to Zr in the periodic table and possess covalent radii slightly smaller than that of Zr, which may facilitate the experimental doping or surface decoration of ZrS_2_. Moreover, recent experimental studies have demonstrated that Sc-, Ti-, and V-doped MoS_2_ exhibit exceptional performance in photocatalytic nitrogen reduction [47], giant photoluminescence [48], and electrocatalytic hydrogen generation [49], respectively, motivating the hypothesis that these elements may also enhance gas adsorption upon decorating ZrS_2_. We comprehensively analyze the influence of different adatom species on adsorption strength, charge transfer, geometric configuration, and potential dissociative adsorption behavior. In addition, binding energies, adsorption energies, density of states (DOS), crystal orbital Hamilton population (COHP), and electron localization function (ELF) analyses are performed to elucidate the underlying interaction mechanisms. Our results reveal that decoration with Sc, Ti, and V significantly enhances the chemisorption of NO, NO_2_, and SO_2_ on ZrS_2_, demonstrating excellent gas capture performance and highlighting single-atom–decorated 1T-ZrS_2_ as a promising gas scavenger for environmental remediation.

## 2. Computational Details

**Figure 1:**
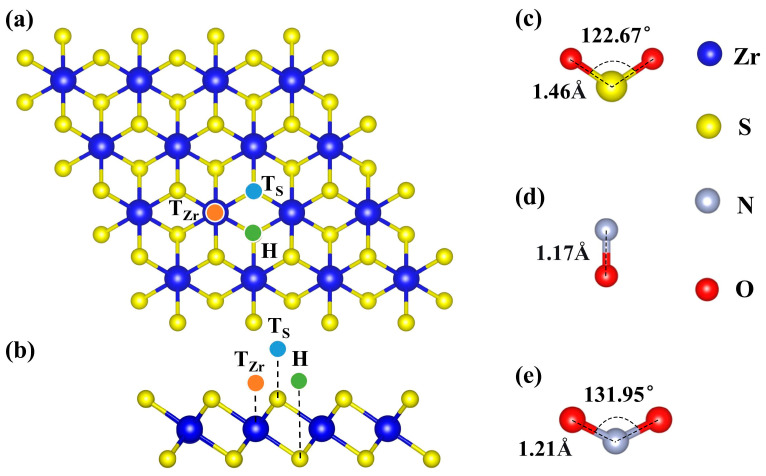
Optimized structures. (**a**,**b**) top and side views of initial ZrS_2_ monolayer, respectively, and (**c**–**e**) Gas molecules SO_2_, NO, and NO_2_, respectively.

All density functional theory (DFT) calculations were performed using the Vienna ab initio Simulation Package (VASP5.4.4) [50,51,52,53]. The generalized gradient approximation (GGA) with the Perdew–Burke–Ernzerhof (PBE) functional was employed to describe the exchange-correlation energy [54,55]. Spin polarization was included in all calculations. The DFT-D3 method was applied to account for van der Waals interactions between the ZrS_2_ monolayer and the adsorbed gas molecules in the transition-metal-decorated systems [56]. The plane-wave cutoff energy was set to 600 eV. In the subsequent calculations, a 4 × 4 × 1 ZrS_2_ supercell containing 16 Zr atoms and 32 S atoms was adopted, as shown in Figure 1a. The Brillouin zone was sampled using a 3 × 3 × 1 *Γ*-centered k-point mesh [57], while a denser 6 × 6 × 1 k-point mesh was employed for the density of states (DOS) calculations to improve accuracy. The band structure and density of states of pristine ZrS_2_ were calculated, showing a band gap of 1.21 eV with the GGA functional and 1.8 eV with the HSE06 hybrid functional, consistent with literature reports [58,59]. To avoid spurious interactions between periodic images, a vacuum spacing of 20 Å was introduced. Geometry optimization was considered converged when the maximum Hellmann-Feynman force on any atom fell below 0.01 eV/Å. During self-consistent-field (SCF) and electronic-property calculations, the energy convergence threshold was set to 1 × 10^−6^ eV.

To identify the most stable adsorption configuration, the binding energy (*E*_bind_) of a transition metal (TM) adatom on the ZrS_2_ surface was calculated using the following equation:

(1) $$E_{bind}=E_{TM-ZrS_{2}}-E_{TM}-E_{ZrS_{2}}$$

where $E_{TM-ZrS_{2}}$, $E_{TM}$ and $E_{ZrS_{2}}$ are the total energy of TM-ZrS_2_ monolayer, TM atoms, and intrinsic ZrS_2_ monolayer, respectively. In addition, *E*_ads_ was estimated to describe the adsorption between gas molecules and substrate, and the formula is as follows:

(2) $$E_{ads}=E_{gas-substrate}-E_{gas}-E_{substrate}$$

where *E*_gas-substrate_, *E*_gas_, and *E*_substrate_ represent the total energy of the adsorbed system, the energy of the gas molecules, and the energy of the substrate, respectively. A negative *E*_ads_ represents an energetically favorable adsorption process, whereas a positive *E*_ads_ corresponds to an energetically unfavorable one. A larger absolute value of the binding energy (*E*_bind_) or adsorption energy (*E*_ads_) indicates stronger binding or adsorption and, consequently, a more stable system.

All subsequent computational post-processing was performed using VASPKIT [60], and the results were visualized with VESTA [61]. To further analyze bonding characteristics, the LOBSTER program [62] was employed to carry out the crystal orbital Hamilton population (COHP) methodology [63]. The Bader charge decomposition was carried out using the efficient algorithm developed by Henkelman and co-workers, in which the core charges were incorporated into the partitions [64,65,66,67]. In this analysis, the electron density was interpolated onto a refined grid of 240 × 240 × 320, and the number of electrons in the vacuum region was confirmed to be zero. To characterize the degree of electron localization and bonding nature, the electron localization function (ELF) [68,69] was employed, and its topological analysis was carried out using the CP2K2022.2 [70] package in conjunction with the Multiwfn3.8 program [71,72]. Additionally, to ensure that the projected density of states (PDOS) analysis was consistent with the crystal-field splitting reported in the previous studies [73,74], the qvasp script [75] was used to rotate the Cartesian coordinates so that each axis aligned along the Zr-S bond direction and corresponded with the octahedral framework surrounding the Zr atoms, thereby ensuring the accuracy of the subsequent electronic-structure analysis.

## 3. Results and Discussion

### 3.1. Geometry and Electronic Structure of the 1T-ZrS_2_ Monolayer Decorated with Sc, Ti, and V Single Atoms

As shown in Figure 1, we optimized the structures of the pristine ZrS_2_ monolayer and of the NO, NO_2_, and SO_2_ molecules. The optimized results indicate that the N-O bond length in the NO molecule is 1.16 Å, while both NO_2_ and SO_2_ adopt a bent (V-shaped) geometry, with N-O and S-O bond lengths of 1.22 Å and 1.48 Å, and O-N-O and O-S-O bond angles of 134.56° and 117.24°, respectively, consistent with previous reports [40,76,77]. The optimized lattice parameters of the ZrS_2_ monolayer are *a* = *b* = 3.69 Å, with a Zr-S bond length of 2.58 Å, in good agreement with prior studies [78,79]. Three representative sites were considered as candidate decoration sites: atop Zr (T_Zr_), atop S (T_S_), and the hollow site (H), as shown in Figure 1a,b. Previous studies (Table 1) have shown that the adsorption of these three gases on pristine ZrS_2_ is relatively weak [44,77,80], which is unfavorable for gas sensing or capture. Accordingly, we next systematically investigated ZrS_2_ decorated with single TM atoms to enhance adsorption strength.

Accordingly, we assessed Sc-, Ti-, and V-decoration of 1T-ZrS_2_ across three candidate adsorption sites (Figure 1) and examined their effects on atomic geometry and electronic properties. The results show that when a TM atom is placed at the sulfur top site (T_S_ site), it relaxes to a nearby hollow site (H site), indicating that this configuration is energetically unstable. Therefore, subsequent analyses focused on the Zr top site (T_Zr_ site) and the hollow site (H site). As summarized in Table 2, all three transition metals prefer the hollow site, where the adsorption energy is the lowest (most negative), corroborating that the H site is the most stable adsorption site on the ZrS_2_ monolayer. This conclusion is consistent with previous theoretical studies [73,74].

The dynamical stability of all TM-decorated ZrS_2_ systems was evaluated by calculating their phonon dispersion relations. As shown in Figure S1, using the Sc-decorated ZrS_2_ system as a representative case, no imaginary frequencies were observed across the Brillouin zone, indicating that the hollow-site configuration is dynamically stable. These results further support selecting the hollow site as the most stable configuration for subsequent structural and electronic analyses.

**Figure 2:**
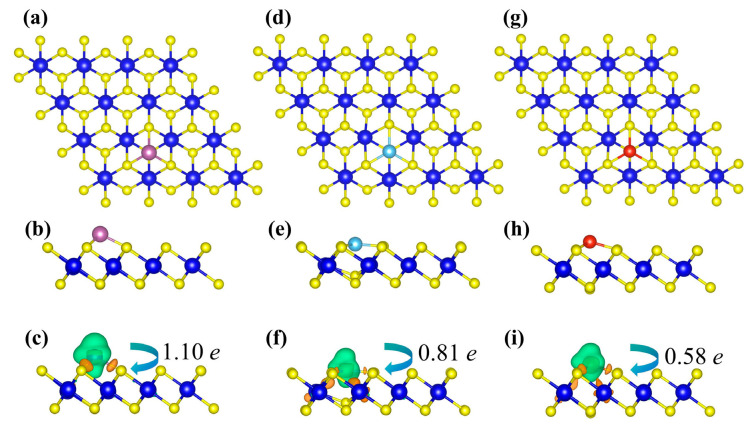
Panels (**a**,**d**,**g**) and (**b**,**e**,**h**) show the top and side views of the Sc-, Ti-, and V-decorated ZrS_2_ systems, respectively. Panels (**c**,**f**,**i**) present the corresponding charge density difference diagrams. In these diagrams, orange and green isosurfaces denote charge accumulation and charge depletion, respectively, with corresponding isovalues of 0.007 e/Å^3^, 0.009 e/Å^3^, and 0.008 e/Å^3^ for the Sc-, Ti-, and V-ZrS_2_ systems.

Figure 2 shows the optimized structures of TM-decorated ZrS_2_ monolayers at the hollow (H) sites, corresponding to the most stable adsorption sites discussed above. Upon TM decoration, only minor distortions of the hexagonal lattice are observed, and each TM atom forms stable TM-S bonds with neighboring S atoms. The bond lengths between the TM dopant atoms (Sc, Ti, and V) and their nearest neighboring S atoms are 2.38 Å, 2.29 Å, and 2.25 Å, respectively. Additionally, the Zr-S bonds adjacent to the dopants are noticeably elongated relative to the pristine value of 2.57 Å, reaching 2.70 Å, 2.79 Å, and 2.65 Å, respectively. Notably, in the Ti-decorated system, the Ti atom slightly embeds into the substrate lattice, causing the underlying S atom to move upward toward Ti. This is consistent with the most negative binding energy among the three, indicating a strong chemical adsorption between the Ti atom and the substrate. Bader analysis shows a net electron transfer from the adatom to ZrS_2_: Sc, Ti, and V donate 1.10 e, 0.81 e, and 0.58 e, respectively. To visualize the charge redistribution, charge density difference (CDD) maps are plotted (Figure 2c,f,i).

To gain a deeper understanding of TM-modified ZrS_2_, we further analyze the PDOS. In the 1T phase, each Zr atom is coordinated by six S atoms, forming an octahedral coordination environment. Under this crystal field, the Zr *d* orbitals split into *e*_g_ and *t*_2g_ manifolds. When a TM atom is adsorbed on the ZrS_2_ surface, it binds to the three nearest-neighbor S atoms. Although the other three coordination positions remain vacant, the TM atom still experiences an approximately octahedral crystal field close to O_h_ symmetry and thus exhibits similar orbital splitting [78,79]. To test this hypothesis, we rotated the Cartesian axes to the local octahedral orientation (*x*, *y* and *z* aligned with the Zr-S bonds) and calculated the PDOS to substantiate this interpretation.

**Figure 3:**
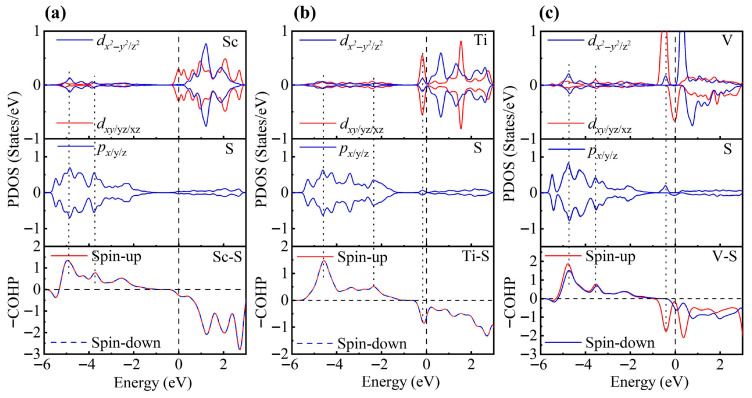
PDOS and COHP of (**a**) Sc-, (**b**) Ti-, and (**c**) V-decorated ZrS_2_ monolayers. DOS showing orbital degeneracy between TM *d*_xy_/*d*_yz_/*d*_xz_ and S *p*_x_/*p*_y_/*p*_z_ orbitals, as well as additional degeneracy of $d_{z^{2\;}}$ and $d_{x^{2}-y^{2}}$ orbitals below the Fermi level.

As shown in Figure 3, the PDOS of the TM-decorated systems shows the characteristic octahedral crystal field splitting: *d*_xy_, *d*_yz_ and *d*_xz_ form a *t*_2g_-like set, whereas $d_{z^{2}}$ and $d_{x^{2}-y^{2}}$ constitute an *e*_g_-like set, as expected. Closer examination of the PDOS for the Sc-decorated system reveals hybridization at −3.85 eV and −4.96 eV. This system exhibits no significant spin splitting, and its total magnetic moment is 0 $\mu_{B}$. In the Ti-decorated system, Ti *d* orbitals and S *p* orbitals exhibited hybridization at −2.47 eV and −4.56 eV, and pronounced hybridization was also present near the Fermi level (around −0.26 eV). The total magnetic moment is 0 $\mu_{B}.$ In contrast, the V-decorated system shows hybridization between V *d* orbitals and S *p* orbitals at −3.55 eV and −4.86 eV, along with pronounced hybridization near the Fermi level. Unlike the Sc- and Ti-decorated systems, the V-decorated system exhibited clear spin polarization, with a total magnetic moment of 0.94 $\mu_{B},$ originating mainly from unpaired V 3*d* electrons. As seen in the figure, hybridization near the Fermi level in TM-decorated systems is dominated by *t*_2g_ orbitals, whereas *e*_g_ orbitals contribute less. Moreover, with increasing *d*-electron count of the TM atom, the occupancy of *d* orbitals near the Fermi level increased, and the hybridization strength of both *t*_2g_ and *e*_g_ orbitals was enhanced.

To gain deeper insight into the interaction between TM atoms and the substrate, we calculated the COHP between TM atoms and neighboring S atoms. In the COHP analysis, the Fermi level is set to 0 eV, with positive values representing bonding states and negative values representing antibonding states. The integrated COHP (ICOHP) below the Fermi level quantifies bond strength, so that more negative values (i.e., larger absolute values) indicate stronger bonding, thereby reflecting the relative strength of atomic interactions. As shown in Figure 3, the V-decorated system exhibits pronounced antibonding states near the Fermi level due to its larger number of *d* electrons, whereas the Sc- and Ti-decorated systems are dominated by bonding states below the Fermi level, with fewer antibonding contributions. Consequently, the V-decorated system has the smallest ICOHP absolute value, corresponding to the weakest adsorption strength. Further analysis reveals that the Sc-decorated system not only shows antibonding states at the Fermi level but also features an additional antibonding peak around −5.46 eV; in contrast, the Ti-decorated system exhibits antibonding states only near the Fermi level. As a result, the Ti-decorated system presents the largest ICOHP absolute value, corresponding to the strongest adsorption and the most significant structural distortion. Specifically, the ICOHP values of the V-, Sc-, and Ti-decorated systems are −4.53 eV, −4.78 eV, and −4.98 eV, respectively, in excellent agreement with the corresponding adsorption energy trends.

### 3.2. Adsorption of NO on TM-Decorated 1T-ZrS_2_ Monolayers

**Figure 4:**
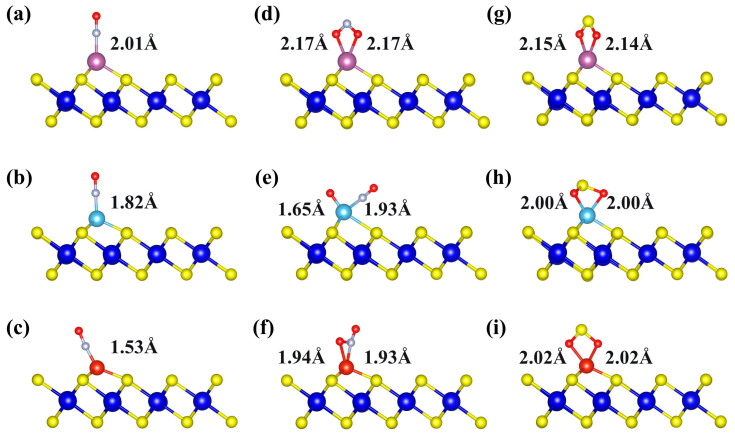
The most stable adsorption structures of three gases on Sc-, Ti-, and V-decorated ZrS_2_ monolayers: (**a**–**c**) NO adsorption on Sc-, Ti-, and V-ZrS_2_, respectively; (**d**–**f**) NO_2_ adsorption on Sc-, Ti-, and V-ZrS_2_, respectively; and (**g**–**i**) SO_2_ adsorption on Sc-, Ti-, and V-ZrS_2_, respectively.

To gain deeper insight into the influence of TM decoration on the gas-adsorption capability, the adsorption behaviors of NO, NO_2_, and SO_2_ molecules on TM-decorated ZrS_2_ monolayers were systematically investigated. As illustrated in Figure 4a–c, the NO molecule binds through the N atom to Sc-, Ti-, and V-decorated ZrS_2_, with TM-N bond lengths of 2.01, 1.82, and 1.53 Å, respectively. After adsorption, the N-O bond length of NO is elongated relative to its optimized value of 1.17 Å, increasing to 1.20, 1.20, and 1.19 Å, respectively. This elongation indicates that the interaction with the TM weakens the intrinsic N–O bond.

**Figure 5:**
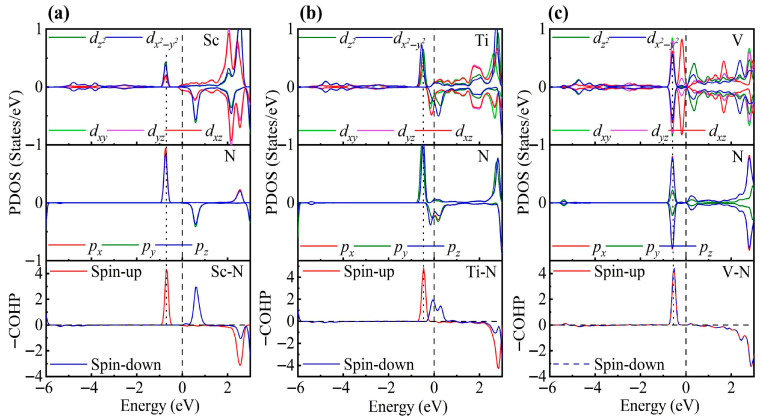
PDOS and COHP for NO adsorption on (**a**) Sc-, (**b**) Ti-, and (**c**) V-decorated ZrS_2_ monolayers. The DOS reveals partial degeneracy of the TM *d*_xy_/*d*_yz_/*d*_xz_ orbitals and the N *p*_x_/*p*_y_/*p*_z_ orbitals.

To elucidate the adsorption mechanism, we analyzed the PDOS. As shown in Figure 5, for NO adsorbed on Sc-ZrS_2_, a pronounced orbital hybridization appears at approximately −0.83 eV below the Fermi level, with contributions from both the *t*_2g_ and *e*_g_ orbitals. The COHP results indicate bonding interactions in this energy region, and the calculated adsorption energy is −2.10 eV. For the Ti-decorated system, in addition to the hybridization between the spin-up orbitals of Ti-*d* and N-*p* states at −0.5 eV, a spin-up bonding state is also present at the Fermi level. This enhances the adsorption, yielding an adsorption energy of −2.47 eV. For the V-decorated system, bonding states appear in both spin up and down channels at approximately −0.76 eV, which further strengthens the adsorption and results in an adsorption energy of −2.89 eV. Moreover, the adsorption strength tends to increase with increasing *d*-electron count, which is likely due to enhanced interaction between the N *p* orbitals and the TM *d* orbitals. Compared with the adsorption energy on the pristine ZrS_2_ monolayer (–0.298 eV) [44], the adsorption energies on TM-decorated ZrS_2_ are significantly enhanced, indicating that TM decoration plays a crucial role in improving the adsorption capability of ZrS_2_.

**Figure 6:**
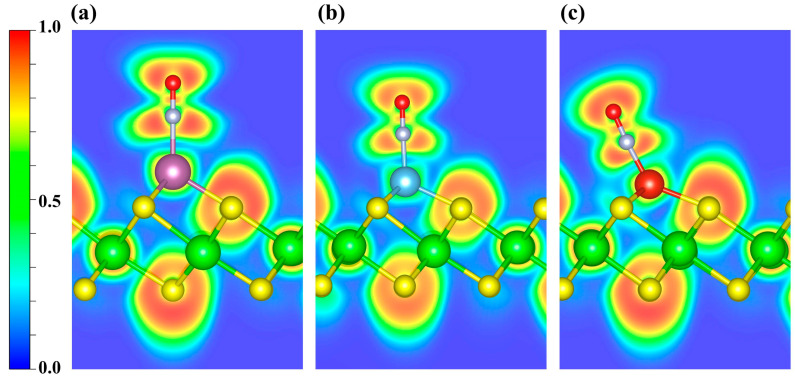
ELF distributions of NO adsorbed on (**a**) Sc-, (**b**) Ti-, and (**c**) V-decorated ZrS_2_ systems. The plots highlight the ELF features around the adsorption sites. Blue and red denote ELF values of 0 and 1, respectively.

As shown in Figure S2, the CDD indicates electron depletion around the transition metal atoms and accumulation on the NO molecule. Further Bader charge analysis reveals charge transfers of 0.47 e, 0.51 e, and 0.48 e to the NO molecule in the Sc-, Ti-, and V-decorated systems, respectively. To elucidate the bonding between NO and the TM atom, we further performed ELF calculations (Figure 6). ELF ranges from 0 to 1: values near 1 denote highly localized electron pairs (covalent bonds or lone pairs); values around 0.5 indicate uniform electron gas; and values near 0 denote very weak localization or very low electron density. As shown, the ELF values are relatively high around the NO molecule, indicating strong electron localization, whereas the regions near the decorated metal atoms exhibit slightly weaker electron localization. Furthermore, the ELF topological analysis (Figure S3a–c) identifies multiple (3, −3) critical points (CPs; ELF attractors) in the N-TM interaction region that lie off the N-TM internuclear line. Moreover, considering the torus-shaped ELF around N (Figure S3d–f), together with the previously discussed PDOS/COHP signatures of orbital hybridization, the NO-TM interaction is likely governed primarily by π-type character.

Furthermore, changes in the magnetic properties were observed upon NO adsorption. For the Sc- and Ti-decorated systems, NO adsorption induces net magnetic moments of 1.96 $\mu_{B}$ and 0.59 $\mu_{B}$, respectively. The reduced net moment in the Ti-decorated system relative to Sc is attributed to partial occupation of spin-down states in the occupied region. For the V-decorated system, NO adsorption reduces the magnetic moment from 0.94 $\mu_{B}$ to a negligible value, rendering the system effectively nonmagnetic.

### 3.3. Adsorption of NO_2_ on TM-Decorated 1T-ZrS_2_ Monolayers

To elucidate the interaction of NO_2_ with Sc-, Ti-, and V-decorated ZrS_2_ monolayers, we first analyzed the optimized adsorption geometries (Figure 4d–f). The results show that NO_2_ is molecularly adsorbed on Sc-ZrS_2_ and V-ZrS_2_, whereas a dissociative adsorption final state is stabilized on Ti-ZrS_2_. In the molecular state, NO_2_ binds in a bidentate fashion: O, O-chelation on Sc and O, N-chelation on V. In contrast, on Ti the adsorbate splits into O and NO fragments: one O forms a Ti-O bond, the N atom of the NO fragment forms a Ti-N bond, and the remaining O stays bonded to N within the NO fragment. Representative bond lengths are Sc-O ≈ 2.17 Å (two bonds), Ti-N = 1.93 Å, Ti-O = 1.65 Å, V-N = 1.92 Å, and V-O = 1.94 Å, consistent with the above configurations.

As summarized in Table 3, NO_2_ binds most strongly on the Ti-decorated system, with an adsorption energy of −3.63 eV, substantially larger in magnitude than on Sc (−3.08 eV) and V (−2.70 eV). As shown in Figure S4, the CDD indicates that during NO_2_ adsorption, electron depletion occurs around the TM atoms, while electron accumulation develops on the molecule, facilitating bond formation between NO_2_ and the TM atom. Further Bader charge analysis reveals that Sc and V transfer 0.67 e and 0.56 e to NO_2_, respectively, whereas Ti exhibits the largest charge transfer of 1.08 e, nearly twice that of V. This pronounced electron redistribution weakens the intramolecular N-O bonds, providing favorable thermodynamic conditions for NO_2_ dissociation. Moreover, the adsorption energies of NO_2_ on all three TM-decorated systems are significantly higher than that on the pristine ZrS_2_ monolayer (−0.16 eV), and the corresponding charge transfers are also markedly larger than that for NO_2_ adsorption on pristine ZrS_2_ (0.04 e) [77], further demonstrating that TM decoration can substantially enhance the adsorption capability of ZrS_2_.

**Figure 7:**
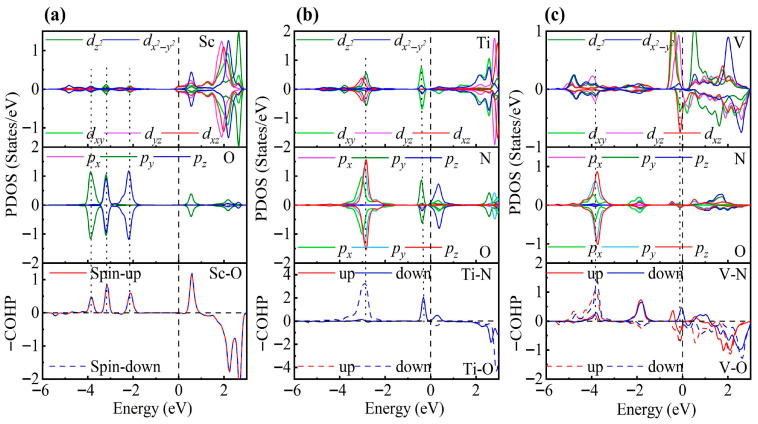
PDOS and COHP for NO_2_ adsorption on (**a**) Sc-, (**b**) Ti-, and (**c**) V-decorated ZrS_2_ monolayers. The DOS reveals partial degeneracy between the TM *d*_xy_/*d*_yz_/*d*_xz_ orbitals and the *p*_x_/*p*_y_/*p*_z_ orbitals of N and O.

Electronic structure analysis clearly reveals that TM decoration significantly enhances the gas adsorption capability of ZrS_2_. As shown in Figure 7, in the Ti-decorated system, the Ti 3*d* states strongly hybridize with the N-*p* and O-*p* orbitals near the Fermi level: the Ti *e*_g_ states hybridize with N-*p_y_* at −0.36 eV, and the Ti $d_{z^{2}}$ state couples with O-*p* at −2.74 eV, forming stable bonding states. Such strong *d*–*p* interactions promote electron transfer from the Ti atom to NO_2_, effectively populating the N-O antibonding orbital, thereby weakening the N-O bond and driving its dissociation. In contrast, the Sc-decorated system exhibits weak *d*-*p* hybridization only at deeper energy levels (−2.05, −3.18, and −3.90 eV), with nearly no contribution near the Fermi level, whereas the V-decorated system shows limited hybridization with O-*p_y/z_* orbitals around −3.87 eV and only weak interaction near the Fermi level. Consequently, NO_2_ remains in a molecular adsorption state on Sc- and V-decorated surfaces but undergoes dissociative adsorption on the Ti-decorated surface.

COHP analysis further substantiates this trend. All three TM-decorated systems enhance the orbital hybridization between NO_2_ and the substrate, but the effect is strongest for Ti. Both Ti-O and Ti-N bonds show pronounced bonding contributions near the Fermi level, with more negative −ICOHP values and larger integrated bonding areas, which together stabilize the dissociative adsorption state. By contrast, Sc exhibits relatively weak bonding peaks, and V even displays antibonding features at the Fermi level, which weakens the metal-ligand interactions. Mechanistically, the cooperative Ti-O/Ti-N bonding channels facilitate electron transfer from the intrinsic N-O bonding orbital to the newly formed metal-ligand bonds, thereby weakening the internal N-O interaction and promoting cleavage. For Sc and V, insufficient *d*-*p* overlap or higher *d*-orbital occupation prevents the formation of such strong multicenter bonding.

**Figure 8:**
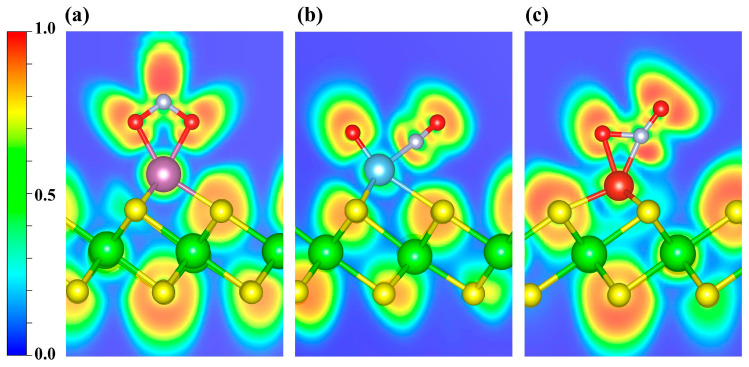
ELF distributions of NO_2_ adsorbed on (**a**) Sc-, (**b**) Ti-, and (**c**) V-decorated ZrS_2_ systems. The plots highlight the ELF features around the adsorption sites. Blue and red denote ELF values of 0 and 1, respectively.

In Figure 8, the ELF maps display pronounced localization around the N and O atoms of NO_2_. To further investigate the bonding interactions, an ELF topological analysis was carried out (Figure S5a–c). It is revealed that the (3, −3) CPs between Sc and the two O atoms lie along their respective internuclear axes (Figure S5a), indicating that both Sc-O bonds are consistent with σ-dominated covalent bonds. However, similar to the case of NO, in the Ti- and V-decorated systems the (3, −3) CPs for TM-O and TM-N lie off the internuclear axis (Figure S5b,c). Taken together with the previously discussed PDOS/COHP evidence for orbital hybridization, the interactions are likely governed primarily by π-type character.

### 3.4. Adsorption of SO_2_ on TM-Decorated 1T-ZrS_2_ Monolayers

As shown in Figure 4g–i, SO_2_ molecules are stably adsorbed on Sc-, Ti- and V-decorated ZrS_2_ monolayers via bonds formed between the two oxygen atoms and the TM atoms. The bond lengths are 2.15 Å and 2.14 Å for Sc-O, 2.00 Å for Ti-O, and 2.02 Å for V-O, indicating a gradual shortening as the atomic number of the TM atom increases. Conventionally, shorter bond lengths are expected to correspond to stronger adsorption. However, the adsorption energies show the opposite trend, with the absolute values decreasing in sequence: Sc, −2.28 eV; Ti, −2.21 eV; V, −1.63 eV. This finding indicates that bond-length reduction alone does not determine adsorption strength, and the adsorption behavior is primarily governed by the electronic structure and orbital interactions of the system.

The CDD and Bader charge analysis further indicate that transition metal modification significantly enhances the adsorption of SO_2_ on the ZrS_2_ surface (Figure S6). During adsorption, the CDD shows electron depletion around the transition metal atoms, while electron accumulation occurs on the SO_2_ molecule, resulting in a stable chemical interaction between the molecule and the TM atom. Bader charge analysis reveals that 0.71 e and 0.73 e are transferred to SO_2_ in the Sc- and Ti-decorated systems, respectively, while the charge transfer is slightly lower in the V-decorated system, at 0.63 e. Consistently, the ICOHP calculations show that the Ti-O interaction is the strongest (−3.24 eV), while the interactions in the Sc and V systems are relatively weaker (−2.54 eV and −2.61 eV, respectively), as listed in Table 3. In contrast, the adsorption energy of SO_2_ on pristine ZrS_2_ is only −0.52 eV, with a charge transfer of merely 0.01 e [77], significantly lower than those of the TM-modified systems.

**Figure 9:**
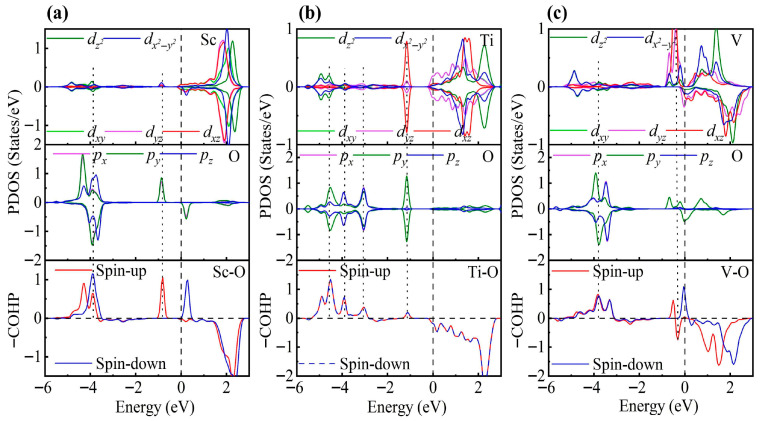
PDOS and COHP for SO_2_ adsorption on (**a**) Sc-, (**b**) Ti-, and (**c**) V-decorated ZrS_2_ monolayers. The DOS reveals partial degeneracy of the TM *d*_xy_/*d*_yz_/*d*_xz_ orbitals and the O *p*_x_/*p*_y_/*p*_z_ orbitals.

The PDOS and COHP analyses further support this conclusion (Figure 9). In the Sc-modified system, a pronounced hybridization between Sc *d* and O *p_y_* orbitals is observed at −0.86 eV, corresponding to a spin-up bonding state, while at −3.90 eV, Sc *d* and O *p* orbitals also exhibit significant hybridization, forming both spin-up and spin-down bonding states. The absence of notable antibonding states near the Fermi level ensures the stability of adsorption. In the Ti-modified system, Ti *t*_2g_ orbitals hybridize with O *p* orbitals at −1.05 eV, and stronger Ti *d*-O *p* hybridizations are observed at −3.08 eV, −3.91 eV, and −4.45 eV, with the largest COHP area at −4.45 eV. Despite the presence of weak antibonding states near the Fermi level, the bonding states dominate, resulting in strong adsorption. In contrast, the V-modified system exhibits pronounced occupation of antibonding states near the Fermi level, mainly involving *t*_2g_ orbitals, with the strongest V *d*-O *p* hybridization at −3.82 eV. The increased d-electron count reduces the covalent contribution, explaining the significantly lower adsorption energy compared to the Sc- and Ti-modified systems.

**Figure 10:**
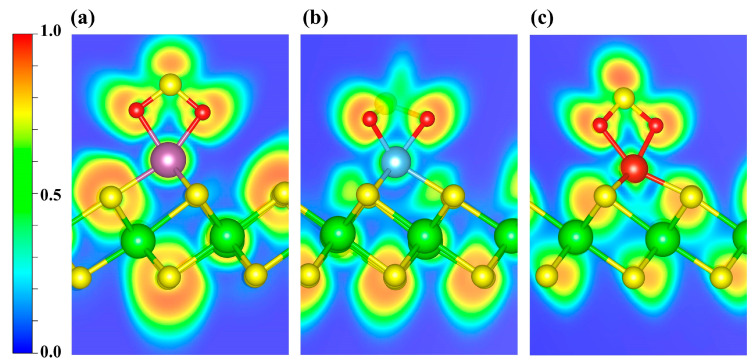
ELF distributions of SO_2_ adsorbed on (**a**) Sc-, (**b**) Ti-, and (**c**) V-decorated ZrS_2_ systems. The plots highlight the ELF features around the adsorption sites. Blue and red denote ELF values of 0 and 1, respectively.

Figure 10 shows the ELF maps for SO_2_ adsorbed on Sc-, Ti-, and V-decorated surfaces, in which the regions around the S and O atoms exhibit pronounced electron localization. An ELF topological analysis (Figure S7a–c) shows that, in the Sc- and V-decorated systems, the (3, −3) CPs lie along the TM-O bond direction (Figure S7a,c), which is consistent with a predominantly σ-type covalent character. In contrast, for SO_2_ adsorption on the Ti-decorated ZrS_2_, the (3, −3) CPs are located outside the bonding axis (Figure S7b). However, given the relatively strong electron localization in the region between Ti and O (Figure 10b and Figure S7e), together with the PDOS/COHP analyses discussed above, we infer that a covalent component also contributes to the Ti-O interaction.

Notably, SO_2_ adsorption modifies the bonding characteristics and significantly affects the magnetic properties of the systems. For Sc-decorated ZrS_2_, which is nonmagnetic prior to adsorption, a magnetic moment of 0.97 $\mu_{B}$ appears after SO_2_ adsorption (Table 3). DOS analysis indicates that the observed moment primarily arises from the SO_2_ molecule, as the O *s* and *p_y_* orbitals near the Fermi level show preferential occupation by spin up electrons, inducing local spin polarization. In contrast, V decorated ZrS_2_ has a magnetic moment of 0.94$\;\mu_{B}$ before adsorption, which increases markedly to 2.96 $\mu_{B}$ after SO_2_ adsorption. The DOS results indicate that this enhancement mainly arises from strong hybridization between V *d_xz_* and *d_yz_* orbitals and O *p_y_* orbitals. This observation further confirms that the strong interaction between SO_2_ and transition metal decorated ZrS_2_ is crucial for tuning the magnetic properties of the system.

### 3.5. Gas Sensing Properties of TM-Decorated 1T-ZrS_2_ Monolayers

**Figure 11:**
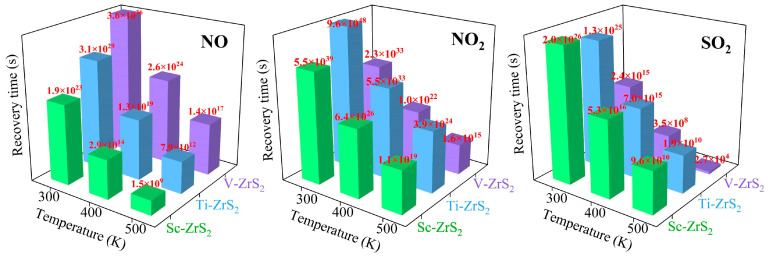
The calculated recovery time (τ) for the desorption of NO, NO_2_, and SO_2_ molecules from Sc-, Ti-, and V-decorated ZrS_2_ monolayers at various temperatures (300 K to 500 K).

The recovery time is an important parameter for gas sensors, which is directly related to the adsorption properties of the material and the sensor’s reusability [81]. The recovery time affects the sensor response speed and also determines its stability and reliability in practical applications. As shown in Figure 11, ZrS_2_ monolayers decorated with TM (Sc, Ti and V) show significantly different recovery times for NO, NO_2_ and SO_2_. The recovery time (τ) was estimated using the van’t Hoff-Arrhenius relation within the framework of transition state theory, as expressed by [82]:

(3) $$\tau ={\nu_{0}}^{-1}e^{\left(-E_{ads}/k_{B}T\right)}$$

where *ν*_0_ is the attempt frequency (typically on the order of 1 × 10^12^ s^−1^ [83]), *E*_ads_ is the adsorption energy, *k*_B_ is the Boltzmann constant (8.617 × 10^−5^ eV·K^−1^), and *T* is the absolute temperature. Here, the recovery times are calculated at three different temperatures: 300 K, 400 K, and 500 K.

The computational results show that at room temperature (300 K) the shortest recovery time calculated for NO desorption from TM decorated ZrS_2_ is 1.9 × 10^23^ s, which is a consequence of the high adsorption energy of NO on the substrate (see Table 3). This result indicates that TM decorated ZrS_2_ can effectively capture NO, but NO is difficult to desorb from this substrate within a short time, which makes the material unsuitable for reversible gas sensing in typical sensor applications. Raising the operating temperature reduces the recovery time; however, even at 400 K and 500 K the desorption remains slow compared with time scales relevant for practical sensing.

For NO_2_ and SO_2_, the shortest recovery times are observed in the V-decorated ZrS_2_ system at 500 K, being 1.6 × 10^15^ s and 2.7 × 10^4^ s, respectively; these values correspond approximately to 5.07 × 10^7^ years for NO_2_ and 7.5 h for SO_2_. Such long recovery times, particularly the extremely long value for NO_2_, indicate that V-decorated ZrS_2_ is unsuitable for reversible gas sensing but may have potential for gas capture or storage applications.

It is worth noting that using the desorption barrier instead of the adsorption energy may yield a more rigorous estimation of recovery time. However, for the systems studied here, the desorption barrier obtained via CI-NEB and the adsorption energy differ by only about 0.1 eV. A similar difference has been reported for CO adsorption on Pt(111) [84]. Therefore, substituting the desorption barrier into the van’t Hoff-Arrhenius relation does not alter the overall conclusions regarding the gas-capture capability of ZrS_2_. Consequently, employing the adsorption energy as an approximation for the desorption barrier is a reasonable and widely adopted approach for estimating recovery behavior.

Compared with the adsorption of NO, NO_2_, and SO_2_ on pristine ZrS_2_, the recovery times of these gases on the transition-metal-decorated structures are significantly prolonged (see Table S1), indicating that the gas desorption process becomes more difficult after decoration. Overall, TM-decorated ZrS_2_ exhibits predominantly irreversible adsorption and slow desorption for all three gases, resulting in poor reusability and rendering the material unsuitable for reversible gas sensing under typical operating conditions. However, the long recovery times indicate strong adsorption, suggesting that TM-decorated ZrS_2_ has potential for gas capture and environmental remediation. Notably, Ti-decorated ZrS_2_ shows catalytic activity toward NO_2_, where the adsorbed molecule undergoes surface dissociation, implying its potential for catalytic degradation or conversion of harmful gases. Therefore, beyond conventional sensing applications, TM-decorated ZrS_2_ may find use in gas capture, storage, environmental cleanup, and catalytic processes. Future studies should focus on optimizing the adsorption–desorption kinetics to balance strong gas capture with controllable desorption, facilitating practical applications in industrial and environmental contexts.

## 4. Conclusions

In summary, we investigated the adsorption of NO, NO_2_, and SO_2_ on Sc-, Ti-, and V-decorated 1T-ZrS_2_ by first-principles calculations. The decorating metals preferentially occupy the hexagonal hollow site of ZrS_2_ and generate an approximately octahedral coordination environment, giving rise to characteristic *d*-orbital splitting. Transition-metal decoration markedly strengthens gas adsorption; notably, Ti decoration yields the strongest interaction with NO_2_ (*E*_ads_ = −3.63 eV) and drives dissociative adsorption, indicating catalytic activation of NO_2_ on Ti-decorated ZrS_2_. Electronic structure analyses indicate pronounced metal-to-adsorbate electron donation and robust hybridization between metal *d* and molecular *p* orbitals, which together underpin the stability of the adsorbed states. Within the investigated temperature range, Sc-, Ti-, and V-decorated ZrS_2_ exhibit relatively long recovery times for NO, NO_2_, and SO_2_, indicating strong adsorption stability and high gas-capture capability. From a practical perspective, Sc, Ti, and V are earth-abundant and less toxic compared with certain heavy metals, making them promising candidates for environmentally sustainable modification of ZrS_2_. Nevertheless, future work should assess their environmental stability before large-scale applications.

## Figures and Tables

**Table 1:** Adsorption energies (*E*_ads_) and charge transfer (*Q*_t_) of three characteristic gases on ZrS_2_ monolayer.

System	Gas Molecule	*E*_ads_ (eV)	*Q*_t_ (e)	Ref.
ZrS_2_	NO	−0.298	-	[44]
ZrS_2_	NO_2_	−0.16	0.04	[77]
ZrS_2_	SO_2_	−0.52	0.01	[77]

**Table 2:** Adsorption positions (T_Zr_: TM adsorbed above a Zr atom, hollow: TM atom adsorbed above the lower-layer S atom), distance between the TM atom (TM = Sc, Ti, V) and its nearest S atom (*d*), binding energies (*E*_bind_), Bader charge transfer (*Q*_t_), and magnetic moments (*M*_tot_) of TM-decorated ZrS_2_.

System	Adsorption Position	*d* (Å)	*E*_bind_ (eV)	*Q*_t_ (e)	$M_{\mathrm{tot}}(\mu_{B}$)
Sc-ZrS_2_	T_Zr_	2.42	−4.82	1.25	0.39
	hollow	2.38	−5.16	1.10	0
Ti-ZrS_2_	T_Zr_	2.31	−4.90	0.94	0
	hollow	2.29	−5.35	0.81	0
V-ZrS_2_	T_Zr_	2.30	−3.71	0.81	2.18
	hollow	2.25	−4.16	0.58	0.94

**Table 3:** Adsorption energy (*E*_ads_), charge transfer (*Q*ₜ), adsorption distance (*D*), gas molecular bond length (*d*), total magnetic moment (*M*ₜₒₜ), and ICOHP for the adsorption of three representative gases (NO, NO_2_, and SO_2_) on Sc-, Ti-, and V-decorated ZrS_2_ monolayers.

Gas Molecule	Atom	*E*_ads_ (eV)	*Q*_t_ (e)	*D* (Å)	*d* (Å)	$M_{\mathrm{tot}}\;(\mu_{B}$)	ICOHP (eV)
NO	Sc	−2.10	0.47	2.01	1.20	1.96	−2.17
	Ti	−2.47	0.51	1.82	1.20	0.59	−3.16
	V	−2.89	0.48	1.53	1.19	0	−3.30
NO_2_	Sc	−3.08	0.67	2.17	1.29	0	−2.32
	Ti	−3.63	1.08	1.65/1.93	1.17	0.04	−7.00
	V	−2.70	0.56	1.93/1.94	1.38/1.20	1.99	−2.94
SO_2_	Sc	−2.28	0.71	2.14/2.15	1.55	0.97	−2.54
	Ti	−2.21	0.73	2.00	1.61	0	−3.24
	V	−1.63	0.63	2.02	1.56	2.96	−2.61

## Data Availability

The data is available on the request from corresponding author.

## Supplementary Materials

The following supporting information can be downloaded at: https://www.mdpi.com/article/10.3390/nano15211653/s1, Figure S1. The calculated phonon dispersion of Sc-decorated 1T-ZrS_2_, evidencing the dynamical stability of the structure. Figure S2. The top and side views of the charge density difference for NO adsorbed on (a) Sc-, (b) Ti-, and (c) V-decorated ZrS_2_ systems. In these diagrams, orange and green isosurfaces denote charge accumulation and charge depletion, respectively, with an isovalue of 0.008 e/Å^3^. Figure S3. Topological analyses of the ELF for NO adsorption on (a) Sc-, (b) Ti-, and (c) V-decorated ZrS_2_. The purple dots represent (3, −3) critical points (CPs), corresponding to local maxima in the ELF. Only the CPs near the decorated atoms and the gas molecule are shown. The chemical bonds were generated by the analysis software, and some bonding representations may contain visualization errors. (d–f) depict the corresponding 3D isosurface representations of the ELF, where n denotes the isosurface value. Figure S4. The top and side views of the charge density difference for NO_2_ adsorbed on (a) Sc-, (b) Ti-, and (c) V-decorated ZrS_2_ systems. In these diagrams, orange and green isosurfaces denote charge accumulation and charge depletion, respectively, with an isovalue of 0.008 e/Å^3^. Figure S5. Topological analyses of the ELF for NO_2_ adsorption on (a) Sc-, (b) Ti-, and (c) V-decorated ZrS_2_. The purple dots represent (3, −3) critical points (CPs), corresponding to local maxima in the ELF. Only the CPs near the decorated atoms and the gas molecule are shown. The chemical bonds were generated by the analysis software, and some bonding representations may contain visualization errors. (d–f) depict the corresponding 3D isosurface representations of the ELF, where n denotes the isosurface value. Figure S6. The top and side views of the charge density difference for SO_2_ adsorbed on (a) Sc-, (b) Ti-, and (c) V-decorated ZrS_2_ systems. In these diagrams, orange and green isosurfaces denote charge accumulation and charge depletion, respectively, with an isovalue of 0.008 e/Å^3^. Figure S7. Topological analyses of the ELF for SO_2_ adsorption on (a) Sc-, (b) Ti-, and (c) V-decorated ZrS_2_. The purple dots represent (3, −3) critical points (CPs), corresponding to local maxima in the ELF. Only the CPs near the decorated atoms and the gas molecule are shown. The chemical bonds were generated by the analysis software, and some bonding representations may contain visualization errors. (d–f) depict the corresponding 3D isosurface representations of the ELF, where n denotes the isosurface value. Table S1. The recovery times (*τ*) of NO, NO_2_, and SO_2_ adsorbed on pristine ZrS_2_, with *T* denoting the adsorption temperature (300, 400, 500 K).
