# Supplementary material for: 1T-ZrS2 Monolayer Decorated with Sc, Ti, and V Single Atoms: A Potential Gas Scavenger for NOx and SO2
Source: Nanomaterials (Basel). 2025 Oct 29;15(21):1653. doi: 10.3390/nano15211653 (PMC12608791; doi:10.3390/nano15211653)
Supplement: Supplementary file 1 [file nanomaterials-15-01653-s001.zip › nanomaterials-3925905-supplementary.pdf]

Supplementary Material

1T-ZrS_2 Monolayer Decorated with Sc, Ti, and V Single Atoms: A Potential Gas Scavenger for NO_x and SO_2

Xiaoxuan Wang [1,†], Jiaqi Zhang [1,†], Jinjuan Zhang [1,*], Xiaoqing Liu [2], Yuanqi Lin [1], Fangfang Li [1], Guangwei Wang [1,*], Yan Xu [1] and Peng Wang [1,*]

1 College of Electronic and Information Engineering, Shandong University of Science and Technology, Qingdao 266590, China; 202482130011@sdust.edu.cn (X.W.); zhangjq_00@sdust.edu.cn (J.Z.); linyuanqi1129@163.com (Y.L.); lifangfang@sdust.edu.cn (F.L.); xuyan@sdust.edu.cn (Y.X.)

2 School of Mathematical Sciences, Shanghai Jiao Tong University, Shanghai 200240, China; liuxiaoq1994@gmail.com

* Correspondence: zhangjinjuan@sdust.edu.cn (J.Z.); wanggw@sdust.edu.cn (G.W.); phywangp@sdust.edu.cn (P.W.)

† These authors contributed equally to this work.

Table of Contents

Figure S1. Calculated phonon dispersion of Sc-ZrS_2 2

Figure S2. Charge density difference of NO adsorption 2

Figure S3. Topological analysis of the ELF for NO adsorption 3

Figure S4. Charge density difference of NO_2 adsorption 4

Figure S5. Topological analysis of the ELF for NO_2 adsorption 4

Figure S6. Charge density difference of SO_2 adsorption 5

Figure S7. Topological analysis of the ELF for SO_2 adsorption 5

Table S1. Recovery times (τ) of NO, NO_2, and SO_2 on pristine ZrS_2 6

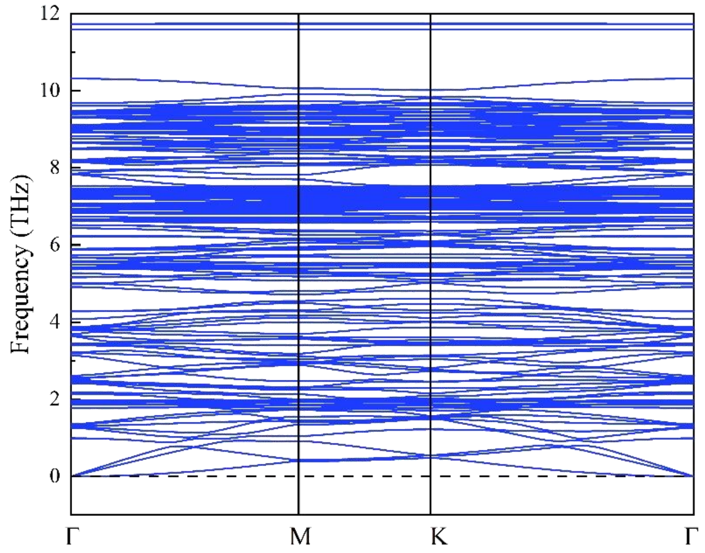

Figure S1. Calculated phonon dispersion of Sc-decorated 1T-ZrS_2, evidencing the dynamical stability of the structure.

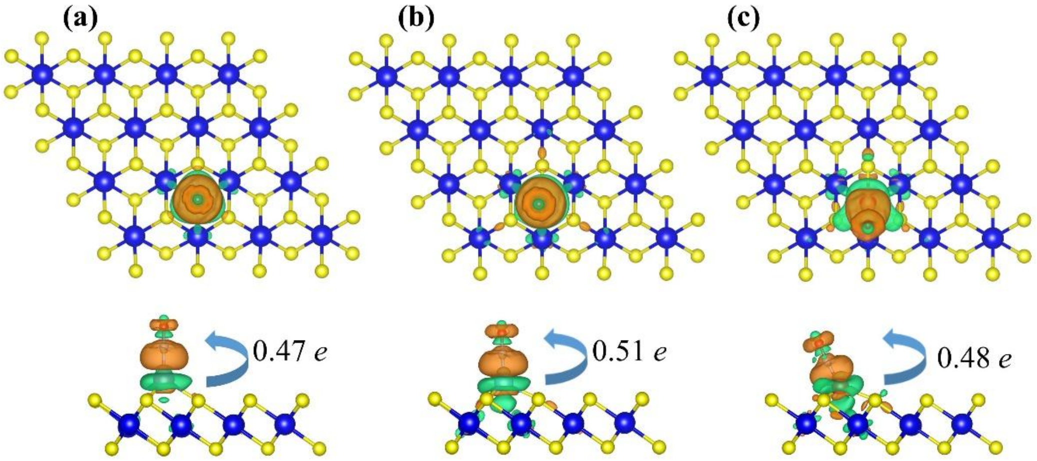

Figure S2. The top and side views of the charge density difference for NO adsorbed on (a) Sc-, (b) Ti-, and (c) V-decorated ZrS_2 systems. In these diagrams, orange and green isosurfaces denote charge accumulation and charge depletion, respectively, with an isovalue of 0.008 e/Å^3.

Since the Multiwfn program used for topological analysis does not support wavefunction files generated from plane-wave-based codes such as VASP, the topological analysis was performed using the CP2K package. The electronic structure calculations were carried out with the DZVP-MOLOPT-SR-GTH basis set in combination with the corresponding GTH pseudopotentials. This setup is expected to provide a reliable description of the electronic density required for accurate evaluation of the ELF and associated critical points (CPs). In the topological analysis, particular attention was paid to the (3, -3) CPs, which correspond to local maxima. For the ELF, such local maxima typically indicate regions associated with covalent bonds, lone pairs, or atomic cores. To further verify the reliability of the topological results, the ELF isosurfaces obtained from VASP calculations were employed for cross-validation. By gradually increasing the ELF isovalue, the local maxima in the ELF distribution were qualitatively identified, which correspond well to the (3, -3) CPs determined from the topological analysis, confirming the correctness of the identified CP positions.

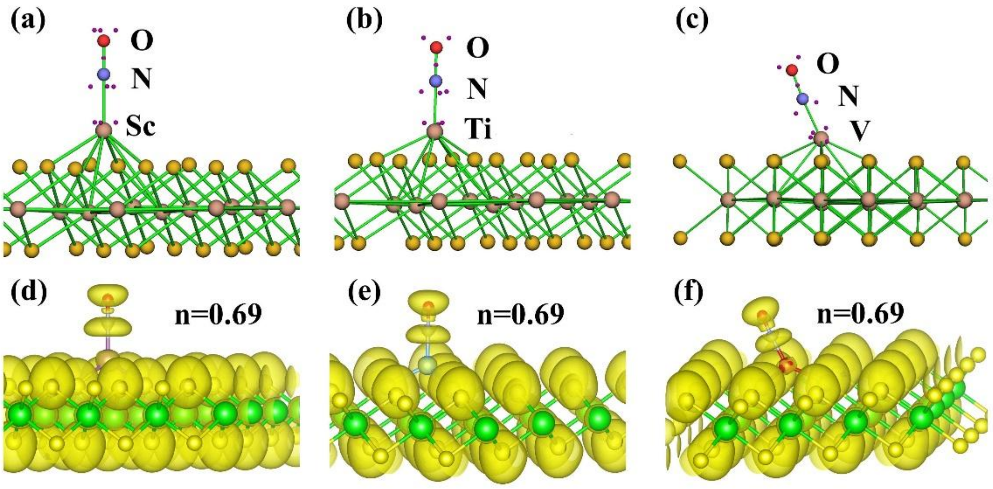

Figure S3. Topological analysis of the ELF for NO adsorption on (a) Sc-, (b) Ti-, and (c) V-decorated ZrS_2. The purple dots represent (3, -3) critical points (CPs), corresponding to local maxima in the ELF. Only the CPs near the decorated atoms and the gas molecule are shown. The chemical bonds were generated by the analysis software, and some bonding representations may contain visualization errors. (d)-(f) depict the corresponding 3D isosurface representations of the ELF, where n denotes the isosurface value.

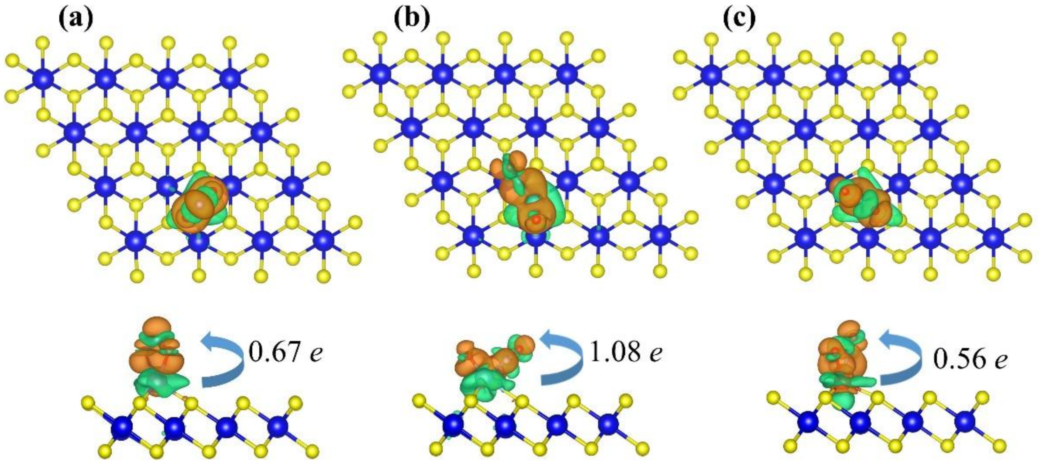

Figure S4. The top and side views of the charge density difference for NO_2 adsorbed on (a) Sc-, (b) Ti-, and (c) V-decorated ZrS_2 systems. In these diagrams, orange and green isosurfaces denote charge accumulation and charge depletion, respectively, with an isovalue of 0.008 $e/Å^3$.

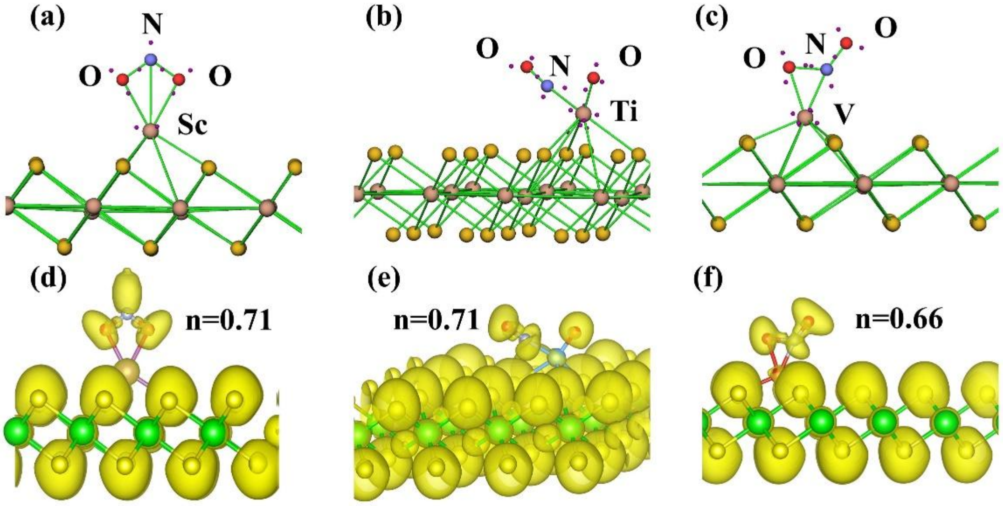

Figure S5. Topological analysis of the ELF for NO_2 adsorption on (a) Sc-, (b) Ti-, and (c) V-decorated ZrS_2. The purple dots represent (3, -3) critical points (CPs), corresponding to local maxima in the ELF. Only the CPs near the decorated atoms and the gas molecule are shown. The chemical bonds were generated by the analysis software, and some bonding representations may contain visualization errors. (d)-(f) depict the corresponding 3D isosurface representations of the ELF, where n denotes the isosurface value.

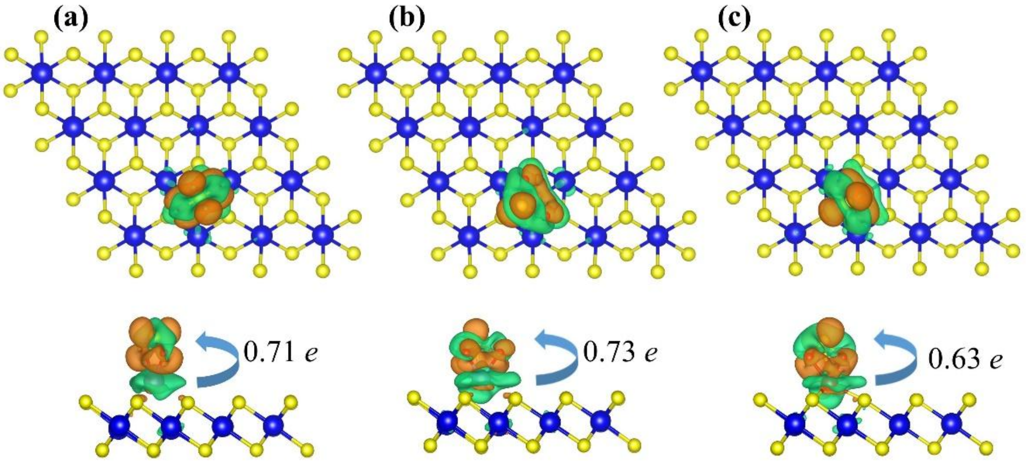

Figure S6. The top and side views of the charge density difference for SO_2 adsorbed on (a) Sc-, (b) Ti-, and (c) V-decorated ZrS_2 systems. In these diagrams, orange and green isosurfaces denote charge accumulation and charge depletion, respectively, with an isovalue of 0.008 $e/Å^3$.

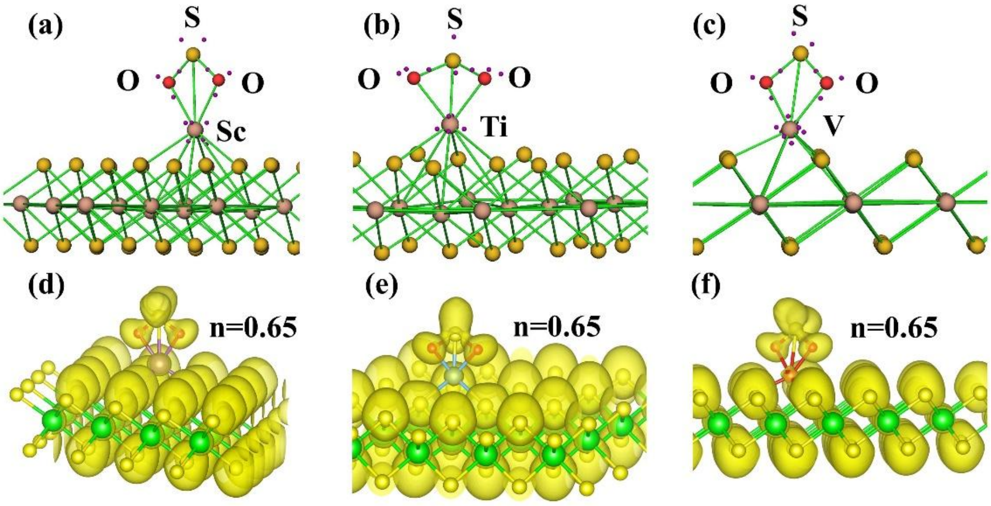

Figure S7. Topological analysis of the ELF for SO_2 adsorption on (a) Sc-, (b) Ti-, and (c) V-decorated ZrS_2. The purple dots represent (3, -3) critical points (CPs), corresponding to local maxima in the ELF. Only the CPs near the decorated atoms and the gas molecule are shown. The chemical bonds were generated by the analysis software, and some bonding representations may contain visualization errors. (d)-(f) depict the corresponding 3D isosurface representations of the ELF, where n denotes the isosurface value.

Table S1. Recovery times (τ) of NO, NO_2, and SO_2 adsorbed on pristine ZrS_2, with T denoting the adsorption temperature (300, 400, 500 K).

System	Gas molecule	T (K)	τ (s)
ZrS_2	NO	300	9.85×10^{18}
		400	1.76×10^{16}
		500	9.91×10^{16}
ZrS_2	NO_2	300	2.05×10^{15}
		400	9.64×10^{15}
		500	2.44×10^{14}
ZrS_2	SO_2	300	1.84×10^{21}
		400	2.81×10^{19}
		500	5.73×10^{18}
